# Why He Was so Lean

**Published:** 1888-12

**Authors:** 


WHY HE WAS SO LEAN.—A lean, misanthropic physician, in a small hamlet, had as his only opponent a handsome robust man. The strife between the two was violent. One day a lady asked the first why he was continually in bad health, whereas the other was so well all the time? "You see, madame," he replied, "the only man who can treat him I am, the only physician whom I can get is he."—*Jour. de Medé. de Paris, according to The Scalpel.*

